# Comparative Genomics of Three *Aspergillus* Strains Reveals Insights into Endophytic Lifestyle and Endophyte-Induced Plant Growth Promotion

**DOI:** 10.3390/jof8070690

**Published:** 2022-06-29

**Authors:** Minyu Jing, Xihui Xu, Jing Peng, Can Li, Hanchao Zhang, Chunlan Lian, Yahua Chen, Zhenguo Shen, Chen Chen

**Affiliations:** 1 College of Life Sciences, Nanjing Agricultural University, Nanjing 210095, China; 2018216009@njau.edu.cn (M.J.); xuxihui@njau.edu.cn (X.X.); 2020116014@njau.edu.cn (J.P.); 2021116040@stu.njau.edu.cn (C.L.); 2019216003@njau.edu.cn (H.Z.); yahuachen@njau.edu.cn (Y.C.); 2 Asian Research Center for Bioresource and Environmental Sciences, Graduate School of Agricultural and Life Sciences, The University of Tokyo, 1-1-1 Midori-cho, Tokyo 188-0002, Japan; lian@anesc.u-tokyo.ac.jp; 3 Jiangsu Collaborative Innovation Center for Solid Organic Waste Resource Utilization, Nanjing Agricultural University, Nanjing 210095, China

**Keywords:** *Aspergillus*, comparative genomics, endophyte, Pi transport, plant growth-promoting

## Abstract

*Aspergillus* includes both plant pathogenic and beneficial fungi. Although endophytes beneficial to plants have high potential for plant growth promotion and improving stress tolerance, studies on endophytic lifestyles and endophyte-plant interactions are still limited. Here, three endophytes belonging to *Aspergillus*, AS31, AS33, and AS42, were isolated. They could successfully colonize rice roots and significantly improved rice growth. The genomes of strains AS31, AS33, and AS42 were sequenced and compared with other *Aspergillus* species covering both pathogens and endophytes. The genomes of AS31, AS33, and AS42 were 36.8, 34.8, and 35.3 Mb, respectively. The endophytic genomes had more genes encoding carbohydrate-active enzymes (CAZymes) and small secreted proteins (SSPs) and secondary metabolism gene clusters involved in indole metabolism than the pathogens. In addition, these endophytes were able to improve Pi (phosphorus) accumulation and transport in rice by inducing the expression of Pi transport genes in rice. Specifically, inoculation with endophytes significantly increased Pi contents in roots at the early stage, while the Pi contents in inoculated shoots were significantly increased at the late stage. Our results not only provide important insights into endophyte-plant interactions but also provide strain and genome resources, paving the way for the agricultural application of *Aspergillus* endophytes.

## 1. Introduction

Microbes play a key role in ecosystems and influence a number of important ecosystem processes, including plant nutrient acquisition, carbon cycling, and soil formation [1,2]. The roots of healthy plants are usually colonized by a rich diversity of microorganisms (such as bacteria and fungi) that regulate plant physiology and development [3,4]. The symbiotic associations of plants with ectomycorrhizal, endomycorrhizal, and endophytic fungi are widespread in agroecosystems, and these fungi enhance host nutrient acquisition and/or tolerance to biotic and abiotic stresses [5,6,7]. Among these associations, endophytic fungi colonize plant tissues and organs without causing symptoms of damage and include a wide variety of filamentous fungi that enhance agricultural productivity [8,9]. One of the most interesting benefits of endophytes is the promotion of host growth. There are multiple mechanisms that directly play important roles in promoting plant growth by endophytes. For example, endophytes produce compounds such as vitamins [10], plant hormones such as indole-3-acetic acid [11] and secondary metabolites [12] to influence plant physiological activities. In addition, plant growth improvement can also be achieved by increasing water availability [13] and/or increasing access to nutrients, such as nitrogen and phosphorus (Pi) [14].

Pi is one of the key mineral elements necessary for plant growth and development, because it is a structural component of nucleic acids and phospholipids and plays important roles in energy transmission, signal transduction, photosynthesis, and respiration [15]. The acquisition of Pi by plant roots is accomplished through its active uptake into the root epidermal and cortical cells via Pi transporters and its transport to shoots via Pi transporters [16]. Thirteen putative high-affinity Pi transport genes belonging to the Pht1 family (*OsPT1*–*OsPT13*) have been identified in the rice genome [17]. Interestingly, the expression of these Pi transport genes might be regulated by microorganisms. For example, *OsPT11* has been shown to be induced in roots by inoculation with arbuscular mycorrhizal fungi [18]. In addition, fungi reportedly play an important role in mobilizing inorganic and organic Pi in the soil and rhizosphere [19,20]. However, the mutualistic interactions between plants and endophytes that induce the Pi-regulatory mechanisms of these endophytes on host plants are still largely unknown.

It has been reported that endophytic fungi could coexist with host plants by modulating sugar pools and plant defenses in their favor, such as the interactions between *Serendipita indica* and *Arabidopsis thaliana* [21]. The plant cell wall is the first and major barrier to infection by fungal pathogens and is a major component of plant biomass. To penetrate plant cells or use plant cells as a carbon source, the genome of plant parasitic fungi encodes many carbohydrate-active enzymes (CAZymes) [22]. In addition, CAZymes have also received special attention due to their importance in the penetration of hosts by phytopathogenic fungi [23]. Compared to parasitic and pathogenic fungi, much less attention has been focused on exploring CAZymes from endophytic fungi. In addition, small secreted proteins (SSPs) are the cornerstone of molecular dialog with host plants, and they act by altering host metabolism and/or defense responses in plant-microbe interactions [24,25]. They alter the processes or structures of host cells, often facilitating the microbial lifestyle [26]. Furthermore, a series of biologically active secondary metabolites could be produced by endophytic fungi driving the formation of mutualistic symbiosis [27,28]. Therefore, identifying the CAZymes, SSPs, and secondary metabolite gene clusters in endophytic fungi could help us to understand the endophyte-plant interactions and the endophytic lifestyles of fungi.

The availability of genome sequences has significantly improved the examination of functional genes in fungi [29]. To date, abundant genomic data have improved our understanding of the evolution of traits involved in symbiosis and parasitism [30,31]. Performing a comparative analysis of multiple genomes to discover the similarities and differences within them can provide insights into fundamental biological questions, such as different nutritional modes of fungi and interactions between plants and fungi [32,33]. Therefore, a comparative genomic analysis of both endophytes and pathogens could be an effective strategy for exploring key traits of endophytic fungi that adapt to plants as habitats [34].

*Aspergillus* is characterized by the unifying feature of the “aspergillum”, an asexual reproductive structure [35]. *Aspergillus* can grow in a wide range of ecological niches, primarily on soil, and some are also able to colonize live plant hosts [36]. To date, more than 300 species have been identified in this genus. For example, some *Aspergillus* species have been reported to be plant pathogens, such as *A. nidulans* (AN), *A. ochraceoroseus* (AO), and *A. tubingensis* (AT) [37,38,39,40,41]. Meanwhile, some *Aspergillus* species are beneficial to plants, such as *A. sydowii* (AS), *A. versicolor* (AV), and *A. awamori* (AA) [42,43,44,45]. Another strain, the engineered fungus *A. puulaauensis* (AP), which was extracted from marine organisms, has been shown to have significant in vitro skin-protective activity against induced oxidative stress [46]. In this study, we isolated three endophytic fungi, AS31, AS33, and AS4, from the healthy roots of *Phytolacca americana* L. The three endophytes could significantly improve rice growth, and their genomes were sequenced. Together with the available pathogenic genomes, the newly sequenced endophytic genomes provided us with an opportunity to explore the mechanisms underlying endophyte-plant interactions and plant growth-promoting properties by comparative genomic analysis.

The main objectives of this study were to test the plant growth-promoting properties of strains AS31, AS33, and AS42 and explore the endophyte-plant interactions between the three strains and rice. We showed that the three isolates AS31, AS33, and AS42 could successfully colonize rice roots. After inoculation with AS31, AS33, and AS42 for seven days, more Pi transport genes were significantly upregulated in rice roots, leading to abundantly accumulated Pi in the roots. In contrast, no significant difference was found for shoots treated with AS31 and AS33. However, 14 days after inoculation, the expression of a large number of Pi transport genes in rice shoots was upregulated, and more Pi was transported to the shoots. In addition, using comparative genomic analysis, we showed that the numbers of CAZymes, SSPs, and indole clusters were significantly higher in endophytes than in pathogens. Our results highlight the importance of Pi uptake and transport in plants regulated by endophytes for plant growth promotion and improve our understanding of endophytic interactions between plants and fungi.

## 2. Materials and Methods

### 2.1. Isolation and Identification of Endophytic Fungi

The healthy and complete roots, stems, and leaves of *P. americana* were washed with sterile water to remove soil and other impurities from the surface. After soaking in 75% ethanol solution (*w*/*v*) for 1 min, these plant tissues were soaked in sodium hypochlorite solution (0.5% active chlorine) for 10 min and then rinsed with sterile water 6 times. The plant tissues were cut into 1 cm pieces and cultivated in potato dextrose agar (PDA) with 100 µg/mL penicillin and 60 µg/mL streptomycin sulfate at 25 °C in the dark. When fungal hyphae grew around the cut of the plant tissue, the tips of the hyphae were transferred to a new PDA for purification and were cultured until a pure fungal colony was obtained.

The three obtained isolates, AS31, AS33, and AS42, were grown in potato dextrose broth (PDB) at 25 °C for 7 days. The hyphae were harvested using sterile filter paper and then ground into fine powder under liquid nitrogen. Genomic DNA was extracted with an E.Z.N.A^®^ Fungal DNA mini kit following the manufacturer’s guidelines (Omega, Darmstadt, Germany). To identify the three isolates, ITS genes were amplified by PCR following the method of Lu et al. [47]. The maximum likelihood (ML) tree implemented in MEGA X [48], the Tamura-Nei model, and 1000 bootstrap replicates were used to perform phylogenetic analysis.

### 2.2. Plant Recolonization Experiments Assessing the Effect of Each Fungal Strain on Plant Growth

Co-cultivations of rice and three isolates (i.e., AS31, AS33, and AS42) were performed according to Lee et al. [49] with modifications. Healthy, plump rice seeds were selected and soaked in 75% ethanol (*w*/*v*) for 1 min and then soaked in sodium hypochlorite solution (1% active chlorine) for 30 min. After being rinsed with sterilized distilled water 6 times, the rice seeds were cultivated in 1/2 Murashige and Skoog (MS) medium [50] for 7 days (in the absence of light) and then transferred to a new 1/2 MS medium. A plug (5 mm diameter) from a PDA plate containing the fungus or a control plug without the fungus was placed in the 1/2 MS medium 0.5 cm away from the root of the rice seedling. The plants were kept at 24/22 °C with a 16 h light/8 h dark photoperiod. At 7 and 14 days after inoculation, plant samples were harvested to measure the shoot height, fresh weight, dry weight, and photosynthetic pigment content of each sample. The contents of Chlorophylls a, Chlorophyll b, and carotenoids were determined according to the method of Lichtenhaler and Wellburn [51]. Each treatment included 6 biological replicates.

A pot experiment was further conducted to test the growth-promoting effect of the three endophytic strains on rice according to Li et al. [52]. The endophytes AS31, AS33, and AS42 were activated in a PDB liquid medium on a shaker at 160 rpm for 72 h at 25 °C. A total of 2 g of mycelia (0.20 g dry weight) were collected, rinsed four times with sterile deionized water, and then inoculated into 200 mL of sterile deionized water to form a fungal suspension. The 7-day-old rice seedlings were transplanted into paddy soils, and each seedling was inoculated with 2 mL of fungal suspension or sterilized distilled water (control). The plants were kept at 24/22 °C with a 16 h light/8 h dark photoperiod. After 60 days of treatment, plant samples were harvested to measure the shoot height, fresh weight, and dry weight. Each treatment included 6 biological replicates.

### 2.3. Determination of Pi Concentrations in Rice Shoots and Roots

The Pi concentrations in the rice roots and shoots were measured using the molybdate blue method [53]. The rice tissues were ground into powder, and 0.4 mL of 10% (*w*/*v*) perchloric acid was added, and the samples were diluted 10-fold with 5% (*w*/*v*) perchloric acid and then placed on ice for 30 min. After the samples were centrifuged at 10,000× *g* for 10 min at 4 °C, 1 mL supernatant was added to 2 mL of working solution [sulfuric acid-ammonium molybdate solution (0.4% ammonium molybdate in 0.5 N H_2_SO_4_): 10% (*w*/*v*) ascorbic acid solution = 6:1]. The mixture was heated at 40 °C for 20 min and then cooled rapidly in an ice bath. The absorbance of the supernatant was recorded at 820 nm. The absorbance values were calibrated to a standard curve generated using a known concentration of Pi.

### 2.4. Fungal Colonization

The colonization site of three *Aspergillus* isolates on rice roots and shoots was monitored using magenta staining [54]. Roots and leaves of rice seedlings inoculated for 7 days and 14 days were cut into 1 cm segments and immersed with 10 mL of KOH solution (100 g/L) in a 90 °C water bath for 90 min. Then, the root or shoot sample was rinsed with deionized water 3 times, and was submerged into 10 mL H_2_O_2_ solution (ω = 30%) for 5 min. After they were further rinsed with deionized water 3 times, the samples were submerged into 10 mL of lactic acid and acidified for 5 min at room temperature. The samples were then stained with acid fuchsin solution (10 mL) for 5 min at room temperature. The samples were soaked in lactic acid glycerol (lactic acid and glycerol in a volume ratio of 1:1) and decolorized overnight at room temperature. The samples were observed under a fluorescence microscope (TI-DH; Nikon, Tokyo, Japan).

The isolation and identification of the three endophytic strains from inoculated rice seedlings were conducted to verify fungal colonization. Rice seedlings were inoculated with three *Aspergillus* isolates for 7 days. Roots and leaves were thoroughly washed with sterile water to remove any external hyphae and then dried with sterile filter paper. For surface disinfection, the root and leaf tissues were soaked in 75% ethanol for 2 min, and in 1% sodium hypochlorite (*v*/*v*) for 5 min, and then washed with sterile deionized water 10 times. Both root and leaf tissues were cut into 0.5 cm^2^ pieces, and then put into malt extract agar medium (MEA) (adding 50 mg/L chloramphenicol to inhibit the growth of bacteria) and incubated on the plate for dark culture at 25 °C. The fungal hyphae that grew from the edge of the tissue incision were collected after 3 days. They were carefully transferred to a fresh PDA medium for purification. For identification, the ITS gene was amplified and sequenced by the method mentioned above.

We further quantified fungal colonization of the root by quantitative PCR (qPCR). Roots of rice seedlings inoculated with strains AS31, AS33, and AS42 for 7 or 14 days were thoroughly washed with sterile water to remove any external hyphae and then dried with sterile filter paper. The DNA of the roots was extracted using a Plant Genomic DNA Kit (Cat#DP305-02, Tiangen Biotech, Beijing, China). The fungal colonization of these root samples was then measured by qPCR. For each sample, qPCR was conducted with two primer pairs, including ITS1F/R (5′-CTTGGTCATTTAGAGGAAGTAA-3′/5′-GCTGCGTTCTTCATCGATGC-3′) targeting the fungal ITS1 sequence, and OsUBQ5-F/R (5′-CTGACGGAGCGTGGTTACTCAT-3′/5′-TCATAGTCCAGGGCGATGTAGG-3′) that targeted *Oryza sativa* UBQ5 (OsUBQ5) gene. Each qPCR was performed by mixing 5 µL of SYBR^®^ Green Premix with 0.2 µL of 10 µM forward primer, 0.2 µL of 10 µM reverse primer, 0.2 µL of 20 µM ROX reference, 3.4 µL of water, and 10 ng of template DNA. The Applied Biosystems StepOnePlusTM Real-Time system was used with the following program: 30 s of denaturation at 95 °C, followed by 40 cycles of 5 s at 95 °C and 30 s at 60 °C. The fungal colonization index was calculated for each sample using the following formula [55]: Index = 2^−Cq(ITS1)/Cq(UBQ5)^.

### 2.5. RNA Extraction and Gene Expression Analysis

To explore the expression pattern of Pi transport genes in rice under inoculation with the three endophytes, the roots and shoots of rice inoculated with fungi for 7 and 14 days were chosen for quantitative real-time PCR (qRT-PCR) analysis. The un-inoculated rice was used as a control. Total RNA was extracted from the rice roots or shoots using a plant RNA extraction kit (Takara, Dalian, China). The RNA quality was analyzed using an Agilent 2100 Bioanalyzer (Agilent Technologies, Santa Clara, CA, USA) and subsequently quantified using a Nanodrop 2000 spectrophotometer (Thermo Fisher Scientific, Waltham, MA, USA). The RNA was reverse-transcribed using a PrimeScript RT Reagent Kit with gDNA Eraser (Takara, Dalian, China). qRT-PCR was conducted using the Applied Biosystems StepOnePlus Real-Time PCR Instrument (Thermo Fisher Scientific, Waltham, MA, USA). The actin gene was used as an endogenous control. The relative gene expression values were calculated using the 2^–ΔΔCt^ method [56]. All the qRT-PCR runs were performed three times using three biological replicates. The primers used in this analysis are provided in Table S1, and the Ct values are provided in Table S2. The qRT-PCR data (2^–ΔΔCt^) were visualized using the pheatmap package in R v4.1.3.

### 2.6. Whole Genome Sequencing, De Novo Assembly, and Functional Annotation

The genome sequencing of strains AS31, AS33, and AS42 was performed with Illumina NovaSeq 6000 platforms, by using the services provided by Biozeron, Shanghai, China. For each genomic DNA sample, 100-bp paired-end libraries with 150- or 500-bp insert sizes were generated using the TruSeq™ Nano DNA Sample Prep Kit (Illumina, San Diego, CA, USA) according to the manufacturer’s instructions. After the sequencing, the adapters and low-quality reads were removed using Trimmomatic v0.39 [57]. The remaining high-quality reads were assembled with ABySS v2.0.2 [58], and the gaps were filled using GapCloser v1.12 [59]. The assembled genomes were annotated de novo with AUGUSTUS v3.2.3 [60]. The predicted genes were compared with the following databases: NR, Swiss-Prot, COG, KEGG, and GO by BLAST (BLAST+ v2.7.1, E-value ≤ 1 × 10^5^ to obtain functional classifications.

For comparative genomic analysis, the genomes of seven *Aspergillus* species were downloaded from the NCBI database, including (i) strains beneficial for plants [*A. sydowii* (PRJNA721994), *A. versicolor* (PRJNA721993), and *A. awamori* (PRJDB4986)]; (ii) strains pathogenic for plants [*A. nidulans* (PRJNA13961), *A. ochraceoroseus* (PRJNA275128), and *A. tubingensis* (PRJNA645154)]; and (iii) other fungi of *A. puulaauensis* (PRJNA728012). The CAZymes were analyzed using the dbCAN HMMER-based classification system with an E-value < 1 × 10^15^ and coverage > 0.35 [61]. The results were catalyzed by their glycoside hydrolases (GHs), glycosyl transferases (GTs), polysaccharide lyases (PLs), carbohydrate esterases (CEs), carbohydrate-binding modules (CBMs), and auxiliary activities (AAs) as described in the CAZy database. The secretome was analyzed by SECRETOOL [62,63]. The secondary metabolite gene clusters were analyzed using Antismash [64].

### 2.7. Statistical Analysis

GRAPHPAD PRISM v9.0.0, R v4.1.3 and SPSS v22.0 (IBM, Armonk, NY, USA) were used for graphing and statistical analyses. Phenotypic and qPCR data were analyzed using a one-way analysis of variance (ANOVA). Duncan’s test was used for multiple comparison analysis, and a value of *p* < 0.05 was considered to be statistically significant. Principal coordinates analysis (PCoA) was used to show differences in CAZymes and secretomes among the 10 *Aspergillus* genomes. Bray-Curtis dissimilarity indices were calculated based on the numbers of CAZymes and secondary metabolite gene clusters using the “vegdist” function in the vegan package. The PCoA plots were generated using the ggplot2 package.

## 3. Results

### 3.1. Characterization of Three Aspergillus Isolates and Plant Growth-Promoting Properties

**Figure 1:**
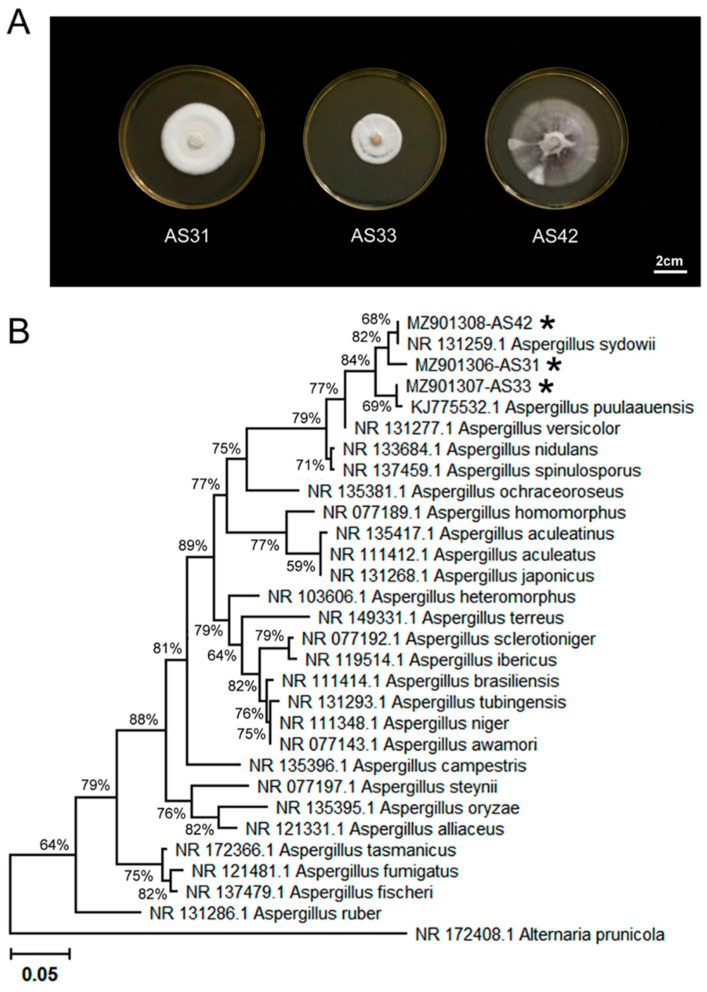
Identification of strains AS31, AS33, and AS42. (**A**) Colony morphology of the three strains isolated from the roots of healthy *Phytolacca americana* L. (**B**) Phylogenetic relationships between the three strains and related species showing the position of strains AS31, AS33, and AS42 within the genus *Aspergillus*. The maximum likelihood (ML) phylogenetic tree is shown. The ML bootstrap values based on 1000 replications are indicated above the branches. Asterisks indicate the strains isolated in this study. Bar, 0.05 substitutions per nucleotide position.

Three fungal strains, AS31, AS33, and AS42, were isolated from healthy pokeweed roots from Nanjing, Jiangsu Province, China (Figure 1A). The ML tree based on ITS sequences showed that strains AS31 and AS42 were clustered together with A. sydowii, which was supported by 68% of the bootstrap value (Figure 1B). The AS33 strain was clustered together with strains of A. puulaauensis, which was supported by 69% of the bootstrap value (Figure 1B). These results showed that the three strains could be assigned to the genus Aspergillus.

**Figure 2:**
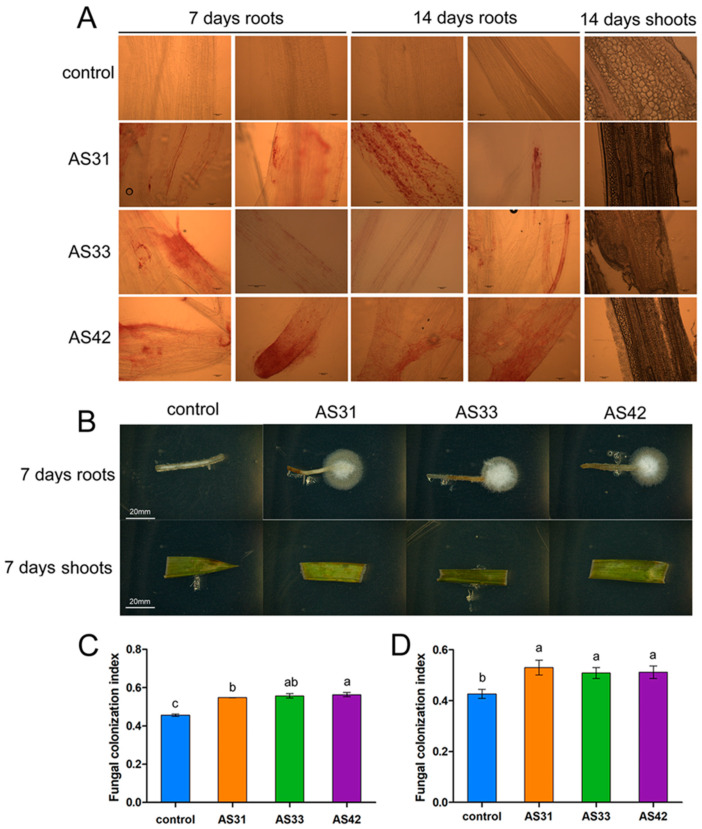
Colonization of strains AS31, AS33, and AS42 in rice roots and shoots after 7 and 14 days of inoculation. (**A**) Magenta staining was used to monitor the inoculation sites on rice roots and shoots. Bars = 100 µm. The three fungi successfully colonized rice roots and produced a large number of hyphae, but these fungi did not colonize rice leaves. (**B**) Isolation of strains AS31, AS33, and AS42 from the inoculated rice roots and shoots. (**C**,**D**) Fungal colonization index at 7 (**C**) days and 14 (**D**) days after inoculation. Data represent the means ± SD of three biological replicates per treatment. Different letters indicate significant differences according to Duncan’s multiple range test. Values of *p*  <  0.05 are considered significant.

Magenta staining was used to monitor the inoculation sites on rice roots and shoots. At 7 days after inoculation, the three fungi successfully colonized rice roots and produced a large number of hyphae (Figure 2A), but these fungi did not colonize rice leaves (Figure 2A). Consistently, the strains AS31, AS33, and AS42 were isolated from the inoculated roots successfully but failed from the shoots (Figure 2B). Besides, the increases in the fungal colonization index for strains AS31, AS33, and AS42 were 24.18%, 19.27%, and 19.96%, respectively, at 14 days after inoculation (Figure 2C,D).

**Figure 3:**
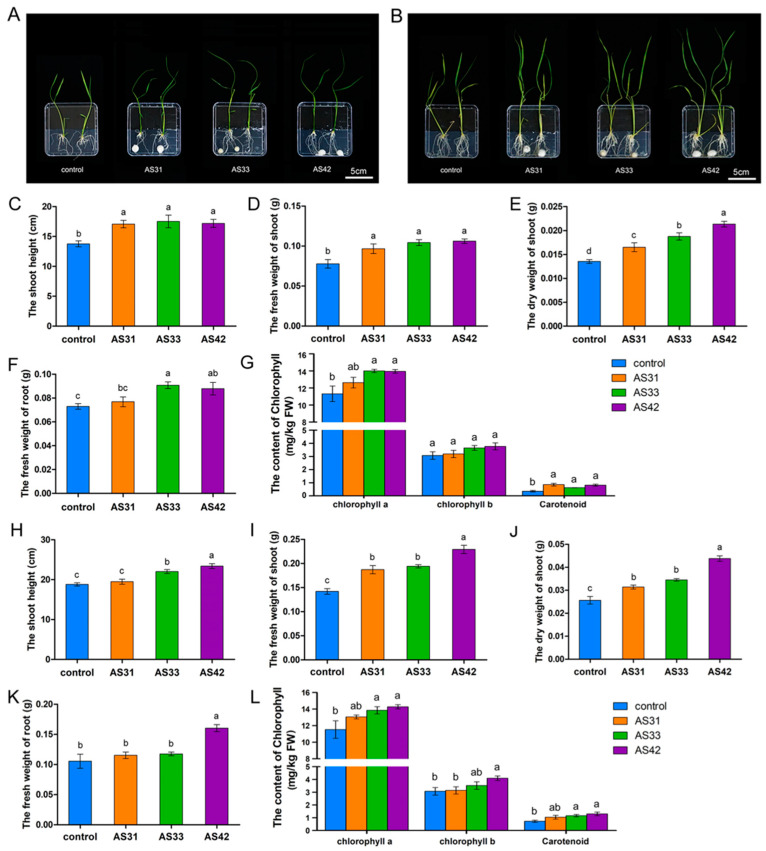
Differential rice responses to inoculation with strains AS31, AS33, or AS42 at the phenotypic level. (**A**,**B**) Promotion of rice growth by the three strains at 7 (**A**) and 14 (**B**) days after inoculation. (**C**–**G**) The shoot height (**C**), fresh weight (**D**), dry weight (**E**), root fresh weight (**F**), and the content of chlorophyll (**G**) of rice inoculated with strains AS31, AS33, or AS42 for 7 days. (**H**–**L**) The shoot height (**H**), fresh weight (**I**), dry weight (**J**), root fresh weight (**K**), and the content of chlorophyll (**L**) of rice inoculated with strains AS31, AS33, or AS42 for 14 days. Data represent the means ± SD of three biological replicates per treatment. Different letters indicate significant differences according to Duncan’s multiple range test. Values of *p*  <  0.05 are considered significant.

Co-cultivation assays of plant and fungal isolates showed that the three strains significantly promoted rice growth and caused no disease on rice (Figure 3). At 7 days after inoculation, all three isolates significantly promoted the growth of rice shoots (Figure 3A,C–E). Specifically, the dry weight of inoculated shoots with strain AS42 was 1.61 times higher than that of the control, and the dry weights of AS31- and AS33-inoculated rice shoots increased by 26.92% and 44.23% compared to the control, respectively (Figure 3D). The weight of inoculated roots was also increased compared to the uninoculated control, especially for roots inoculated with strains AS33 and AS42 (Figure 3F). Meanwhile, the chlorophyll a content of three isolates-inoculated rice shoots increased by 11.59%, 23.69%, and 23.20% compared to the control, respectively (Figure 3G). As well, the carotenoid content of inoculated shoots also significantly increased compared to the control, respectively (Figure 3G).

At 14 days after inoculation, the inoculated shoots showed greater lengths and fresh weights than the uninoculated control (Figure 3B,H–J). The shoot dry weights increased by 22.66%, 34.77%, and 71.09% in AS31-, AS33-, and AS42-inoculated plants compared to the control, respectively (Figure 3J). For the roots, AS42 inoculation increased the root weight, while the other two strains showed little effect on this characteristic (Figure 3K). The treatments of AS33 and AS42 increased the contents of chlorophyll a, chlorophyll b, and carotenoid significantly, while the AS31 treatment increased chlorophyll a and carotenoid contents (Figure 3L). Together, these results showed that strains AS31, AS33, and AS42 could colonize rice roots and form symbiotic interactions with them, promoting rice growth without causing rice diseases.

**Figure 4:**
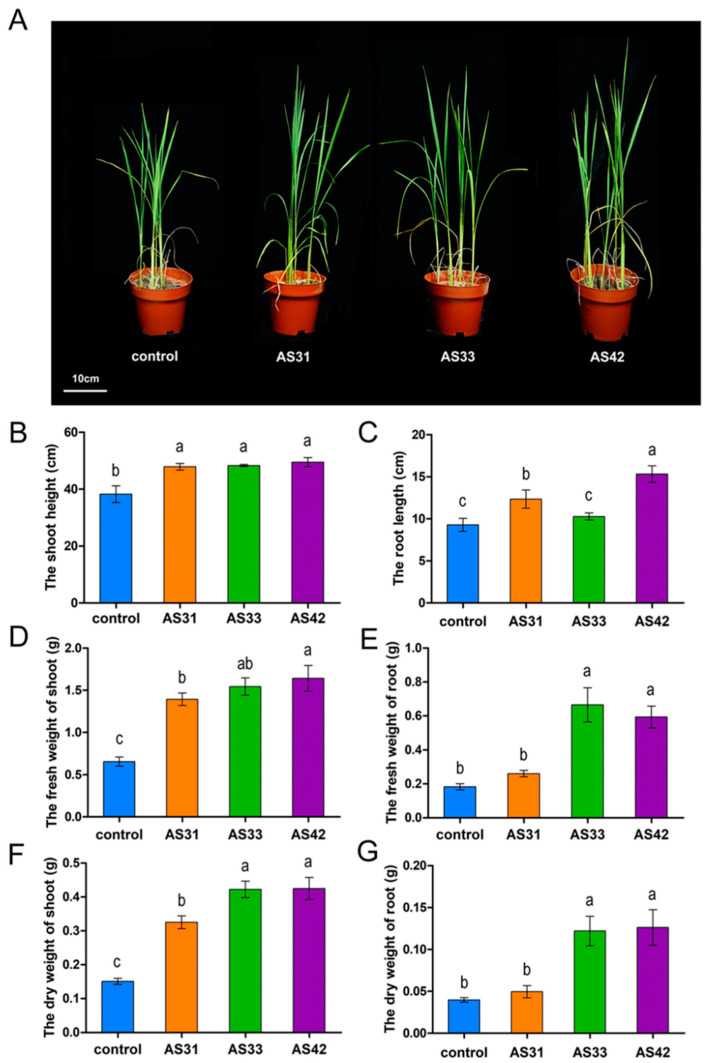
Promotions of rice growth by inoculation of strains AS31, AS33, or AS42 in soils. Differential rice responses to inoculation with strains AS31, AS33, or AS42 in the pot experiments are shown. (**A**) Promotion of rice growth by the three strains at 60 days after inoculation. (**B**–**G**) The shoot height (**B**), fresh weight (**D**), dry weight (**F**), and root length (**C**), root fresh weight (**E**), root dry weight (**G**) of rice inoculated with strains AS31, AS33, or AS42 for 60 days in the pot experiments. Data represent the means ± SD of six biological replicates per treatment. Different letters indicate significant differences according to Duncan’s multiple range test. Values of *p*  <  0.05 are considered significant.

To determine the beneficial effects of endophyte-inoculation on rice plants in soils, we compared the plant biomass of AS31-, AS33- and AS42-inoculated rice with un-inoculated rice by pot experiments. The three strains significantly promoted rice growth for long-term and caused no disease on rice (Figure 4). The shoot heights of rice inoculated with strains AS31, AS33, and AS42 were increased by 25.23%, 26.35%, and 29.48% compared to control, respectively (Figure 4B). Besides, the dry weights of inoculated shoots with strains AS31, AS33, and AS42 were 1.16, 1.80, and 1.82 times higher than that of control, respectively (Figure 4F). Meanwhile, the dry weight of roots increased by 2.07, and 2.18 times in AS33-, and AS42-inoculated plants compared to control, respectively, while the AS31 showed little effect on rice roots (Figure 4G).

### 3.2. Genome Features of Strains AS31, AS33, and AS42

The total lengths of the genomes of AS31, AS33, and AS42 were 36.8, 34.8, and 35.3 Mb, with GC contents of 49.94%, 49.57%, and 50.47%, respectively (Table 1). A total of 12,898, 12,933, and 12,921 genes were identified in the genomes of AS33, AS31, and AS42, of which 213 (1.65%), 174 (1.35%), and 126 (0.97%) genes failed to find a hit in the NR database, respectively (Table 1). Among them, 4644, 4684, and 4612 genes of AS31, AS33, and AS42 showed significant matches (E-value < 1 × 10^5^) in the KEGG database, respectively, which were enriched in 34 KEGG pathways (Figure S1). The most enriched pathway was metabolic pathways (1386, 1469, and 1369 genes for AS31, AS33, and AS42, respectively), followed by biosynthesis of secondary metabolites (541, 535, and 540 genes, respectively). In addition, the top 20 KEGG enriched pathways in the three sequenced genomes were similar (Figure S2). According to the GO database, 4093, 4117, and 4128 predicted genes of AS31, AS33, and AS42, respectively, were annotated (Table 1). These genes were primarily distributed across five functional entries, namely, “metabolic process”, “single-organism process”, “cellular process”, “catalytic activity”, and “binding” (Figure S3). Genome annotation according to different databases (including NR, SwissProt, COG, GO, and KEGG) showed 2698, 2690, and 2676 overlapping genes, accounting for 20.86%, 21.76%, and 20.26% of the total genes in the genomes of AS31, AS33, and AS42, respectively (Figure S4).

### 3.3. Comparative Analysis of CAZymes and SSPs

**Figure 5:**
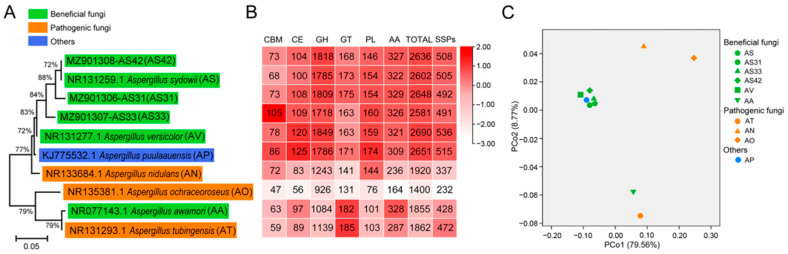
Comparative analysis of CAZyme and secretome encoded in the genomes of AS31, AS33, AS42, and related species. (**A**) Maximum likelihood (ML) phylogenetic tree showing the evolutionary relationships of six beneficial (green) species, three pathogenic (orange) species, and one other (blue) species. The names in brackets refer to the abbreviation of each species. (**B**) Numbers of CAZymes and SSPs encoded in the 10 genomes according to A. CBM, carbohydrate-binding module; CE, carbohydrate esterase; GH, glycoside hydrolase; GT, glycosyl transferase; PL, polysaccharide lyases; AA, auxiliary activity; TOTAL, total number of CAZymes; SSPs, small secreted proteins. (**C**) Principal coordinates analysis (PCoA) of CAZyme and secretome based on gene numbers. The abbreviation of each species is the same as A.

CAZymes have been reported to be essential for fungal bioactivity [33]. For both plant pathogens and endophytes, CAZymes are responsible for degrading host plant cells and establishing colonization [63,64]. To explore the CAZymes of fungi with different nutrition lifestyles, we selected seven Aspergillus strains that had available genomes and were closely related to the three isolated strains for a comparative genomic analysis (Figure 5A; Table S3). The seven selected strains covered both plant pathogenic and beneficial fungi. The phylogenetic analysis showed that the plant growth-promoting fungi AS and AV were clustered together with strains AS31, AS33, and AS42 (Figure 5A).

The AS31, AS33, and AS42 genomes encoded 2648, 2581, and 2636 CAZymes, including 1818, 1718, and 1809 GHs, 168, 163, and 175 GTs, and 104, 109, and 108 CEs, respectively (Figure 5B). Interestingly, the beneficial fungi (three endophytes and AS and AV) had many more CAZymes (>2580) than those in pathogenic fungi (<1920; Figure 5B). In addition, PCoA pointed to distinct clusters of plant beneficial and pathogenic fungi (Figure 5C). Specifically, the fungi beneficial to plant growth were clustered together (except for AA), while the pathogens were more dispersed and had no clusters (Figure 5C). These results showed that strains AS31, AS33, and AS42 exhibited similar patterns of CAZymes with the beneficial fungi compared to pathogenic fungi. Given the functions of CAZymes in carbohydrate utilization, the different numbers of CAZymes among beneficial and pathogenic fungi showed that CAZymes might be important for determining the lifestyles of the tested Aspergillus fungi.

**Figure 6:**
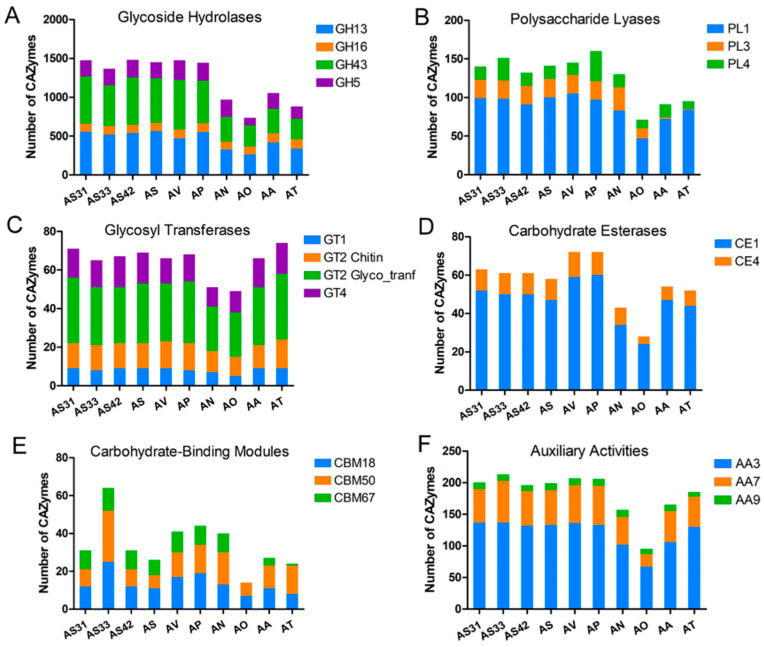
Numbers of genes related to CAZyme from the genomes of AS31, AS33, and AS42. (**A**) numbers of genes dominated in categories of glycoside hydrolase (GH); (**B**) numbers of genes dominated in categories of polysaccharide lyases (PL). (**C**) numbers of genes dominated in categories of glycosyl transferase (GT); (**D**) numbers of genes dominated in categories of carbohydrate esterase (CE); (**E**) numbers of genes dominated in categories of carbohydrate-binding module (CBM); (**F**) numbers of genes dominated in categories of auxiliary activity (AA).

Among these CAZymes, the GH superfamily was more enriched in the beneficial strains (except for AA) compared to the pathogens (Figure 5B). Among the superfamily members, GH5, GH13, GH16, and GH43 accounted for a higher proportion (Figure 6A). In addition to GH, the PL superfamily was also detected in the three genomes of endophytes, with PL1 being the most dominant family, followed by PL3 and PL4 (Figure 6B). GT1, GT2_Chitin, GT2 Glyco_tranf, and GT4 in GTs; CE1 in CEs; CBM18, CBM67, and CBM50 in CBMs; and AA3, AA7, and AA9 in AAs were also highly enriched in the three genomes (Figure 6C–F). Notably, the AA9, GH5, GT1, PL1, and PL3 gene families were found to be critical for endophytic fungal colonization [63,65].

A total of 492, 491, and 508 SSPs were found in the genomes of AS31, AS33, and AS42, respectively (Figure 5B). Similar to the distribution of CAZymes among different types of fungi, fungi beneficial to plants had many more SSPs than pathogens (Figure 5B). The larger number of SSPs in beneficial fungi showed that SSPs might play important roles in the symbiosis of fungal-plant interactions.

### 3.4. Identification of Secondary Metabolism Gene Clusters

The genetic coding possibilities of secondary metabolites, the stimuli produced by secondary metabolites, and special phytotoxins basically determine the microscopic interactions between fungi and host plants [27]. Fungal secondary metabolites might mimic plant effect molecules, such as auxin, gibberellin, and abscisic acid, to obtain nutrients needed for fungal growth and colonization [66]. The secondary metabolites of beneficial microbes have also been shown to be the leading candidates to regulate plant growth promotion [67]. To explore the coding possibilities of secondary metabolites in the three sequenced genomes, the secondary metabolism gene clusters were analyzed and compared with the seven other closely related Aspergillus fungi (Table S4). The results showed that these genomes encode many secondary metabolism gene clusters, such as gene clusters for non-ribosomal polypeptide synthase (NRPS), Type I polyketide synthases (T1PKS), terpenes, and indole (Table S4). In contrast to the greater detection of CAZymes and SSPs in beneficial fungi than in pathogenic fungi, similar (such as AN) or higher amounts (such as AT) of secondary metabolism gene clusters were detected in pathogenic fungi than in beneficial fungi (Table S4). Interestingly, more indole clusters were detected in beneficial fungi than in pathogenic fungi (Table S4), showing the important roles of indole metabolism in the interactions between endophytes and plants. In addition, the genomes of strains AS31, AS33, and AS42 have 14 identical gene clusters encoding metabolites such as naphthopyrone, neosartorin, shearinine D, clavaric acid, and asperthecin (Tables S5–S7), which might be involved in endophyte-plant interactions. For example, asperthecin plays an important role in sexual spore maturation and cellular and metabolic integrity and is involved in successful root colonization [68].

### 3.5. Endophytes Altered Pi transportation and Distribution in Rice by Regulating the Expression of Pi Transport Genes in the Host Plant

**Figure 7:**
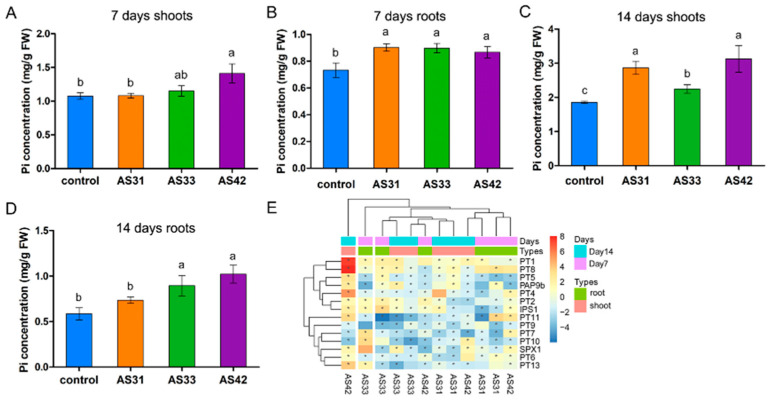
Pi accumulation and expression of Pi transport-related genes in rice in response to the inoculation of strains AS31, AS33, or AS42 after 7 and 14 days. (**A**,**B**) Pi concentrations in shoots (**A**) and roots (**B**) of rice inoculated with each strain after 7 days; (**C**,**D**) Pi concentrations of shoots (**C**) and roots (**D**) of rice inoculated with each strain after 14 days. Different letters indicate significant differences according to Duncan’s multiple range test. Values of *p*  <  0.05 are considered significant. (**E**) heat map showing the expression (log2-fold change of inoculated vs. uninoculated samples) of Pi transport-related genes in both the roots and shoots of rice at 7 and 14 days after inoculation with strains AS31, AS33, or AS42. The gene expression was obtained by quantitative real-time PCR (qRT-PCR), and the primers used in the qRT-PCR are listed in Table S1. Red, increase in gene expressions; blue, decrease in gene expressions. Asterisks indicate significant differences (*p* < 0.05).

Seven days after inoculation with the endophytes AS31, AS33, and AS42, the Pi concentrations in rice roots were significantly increased compared to the control, while the increased Pi concentrations in the shoots were only detected in AS42-inoculated roots (Figure 7A,B). However, the Pi concentrations in both the shoots and roots of rice with inoculations increased significantly compared to the control at 14 days after inoculations, except for the AS31-inoculated roots (Figure 7C,D). Among the three endophytes, rice roots and shoots inoculated with strain AS42 showed the highest Pi concentration compared to those inoculated with the other two strains (Figure 7A–D). These results were consistent with the finding that strain AS42 had the best effect on promoting rice growth (Figure 3).

At 7 days after inoculation, many Pi transport genes were significantly upregulated in rice roots compared to the control (Figure 7E, Table S2). Specifically, under AS31 treatment, PT6, PT8, PAP9b, and PT11 were significantly upregulated in roots compared to the control. Similarly, the expression levels of PT1, PT2, PT7, PT10, IPS1, SPX1, and PT13 were significantly upregulated in AS33-inoculated roots, while PT4, PT7, PT8, and PT13 were significantly upregulated in AS42-inoculated roots. However, only a few genes were upregulated (two, four, and two genes for AS31, AS33, and AS42, respectively) in the shoots at 7 days after inoculation (Figure 7E). These results indicated that at the early stage of colonization, the three endophytes primarily affected the expression of Pi transport genes in roots to alter the uptake and transport of Pi in roots rather than shoots.

By contrast, 14 days after inoculation, the expression patterns of these Pi transport genes changed in both the rice roots and shoots (Figure 7E). For example, a large number of genes were upregulated in the shoots (3, 6, and 11 genes for AS31, AS33, and AS42, respectively), while only a few genes were upregulated in the roots (two, zero, and four genes for AS31, AS33, and AS42, respectively). The results showed that the endophyte-induced effects on Pi uptake and transport were primarily in the shoots compared to the roots at the late stage. Although the upregulation of PT1 (in roots and shoots) and PT8 (in shoots) was detected in rice inoculated with all three endophytes, most upregulated genes were strain-specific; for example, PT6, PT10, and SPX1 were specific to AS42-treated roots, and PT4, PT6, and PT11 were specific to AS42-treated shoots (Figure 7E). These results showed the different expression patterns of Pi transport genes induced by the three endophytes, which might lead to differences in Pi accumulation in rice and thus have different effects on rice growth promotion.

## 4. Discussion

The *Aspergillus* genus includes a large number of species, including both pathogenic and beneficial fungi for plants [35,36]. Among them, species that cause plant disease have been extensively studied, and many of their genomes have been available thus far [35,69]. Although *Aspergillus* species beneficial to plants have high potential for promoting plant growth and improving stress tolerance, studies on endophytic fungal lifestyles and symbiotic interactions between endophytes and plants are still limited. One important hindrance is the relative lack of endophytic fungi belonging to *Aspergillus* and their genomes. In the current study, we isolated three endophytic *Aspergillus* isolates that were able to colonize rice roots and significantly promoted the growth of rice seedling shoots and roots. To explore the beneficial interactions between endophytes and host plants, the genomes of the three endophytes were sequenced and compared to other related strains covering different lifestyles, including both pathogenic and beneficial fungi. We showed that these endophytes were able to regulate Pi accumulation and transport in rice. In addition, more CAZymes, indole clusters, and SSPs were detected in the genomes of these endophytes than in those of pathogenic fungi, showing the importance of these genes in the beneficial interactions between endophytes and plants. Our results not only provide important insights into endophyte-plant interactions but also provide strain and genome resources facilitating the agricultural application of endophytic fungi of *Aspergillus*.

The available Pi content in roots increased significantly 7 days after inoculation with the three endophytes, indicating that strains AS31, AS33, and AS42 affected the absorption and accumulation of Pi in rice. Pi-mediated interactions between plants and endophytes were also detected for other endophytes. For example, the endophytic *Helotiales* fungus F229 has been reported to promote the growth of *Alpinia chinensis* in low-Pi soils and the acquisition of Pi [70]; the fungus *Xylaria regalis* isolated from arborvitae increases the Pi content of seedling tissues [71]; as well, *Phomopsis liquidambari* enhances Pi acquisition and utilization by rice [72]. Interestingly, although no significant difference in Pi content in the shoots was detected at 7 days after inoculation with strains AS31 or AS33 compared to the control, the Pi content in the inoculated shoots was significantly higher than that in the control at 14 days after inoculation with AS31 or AS33. One possible explanation is that Pi is a mobile element in plants that can be transported from roots to young tissues to ensure the growth of young tissues in the late growth stage or under phosphorus-deficient conditions [73]. Together, our results showed that more Pi was transported from the roots to the shoots at 14 days after inoculation compared to the early stage.

To explore the underlying mechanisms of Pi transportation in rice induced by endophytes, the expression of Pi transport genes in rice was further analyzed. We found that at 7 days after inoculation, more Pi transport genes were upregulated in the root-control comparison vs. the shoot-control comparison, which is consistent with the increased Pi contents in roots rather than shoots at the early stage. For example, *OsPT11* was significantly upregulated in AS31- or AS42-inoculated roots but not shoots. Consistently, *OsPT11* has been shown to be specifically induced during root symbiosis with *Glomus intraradices*, and this induction is restricted to the root system [18]. In contrast to strains AS31 and AS42, strain AS33 induced the upregulation of *OsPT10* and *OsSPX1*. The expression of *OsPT10* is specifically induced by Pi starvation and plays a role in Pi uptake [74], while *OsSPX1* regulates signal transduction under Pi starvation and regulates Pi homeostasis [75]. The results showed that strain AS33 regulates the accumulation of Pi in rice with different pathways than strains AS31 and AS42. At 14 days after inoculation, more upregulated Pi transport genes (e.g., *OsPT1* and *OsPT8*) were detected in inoculated shoots than in roots, which is also consistent with the significantly increased Pi contents in inoculated shoots at 14 days after inoculation. Previous studies have shown that *OsPT1* is a key member of the Pht1 family and is involved in the uptake and transport of Pi in rice under Pi-sufficient conditions [76], while *OsPT8* is involved in Pi homeostasis in rice, which is essential for plant growth and development [77]. Together, these results showed that the three endophytes were able to improve Pi accumulation and transport in rice by inducing the expression of Pi transport-related genes in rice.

Comparative genomics could provide new clues to identify a core set of CAZymes associated with the endophytic lifestyle of fungi [55,78]. In this study, we investigated the CAZymes of AS31, AS33, and AS42 and compared them with those of other related fungi. We found that endophytic fungi contained larger amounts of CAZymes than pathogenic fungi. The GH superfamily was the most representative class among the CAZymes in the three endophytic strains, including GH5, GH13, and GH43. GH5 is considered critical for establishing ectomycorrhizal symbiosis and endophytic adaptation [55,78]. GH13 contains thiamine transporter, NAD/NADP-dependent betaine aldehyde dehydrogenase, alpha-amylase, and high-affinity glucose transporter, which are related to nutrient acquisition and metabolism and are essential for symbiotic relationships with plants [79,80]. GH43 is primarily responsible for the hydrolysis of xylan/arabinoxylan and includes β-xylosidase, α-galactosidase, α-L-arabinosidase, and α-arabinofuranosidase [81]. In addition, the AA superfamily was also increased in the three endophytes compared to the pathogens, such as AA3, which is important for cellobiose dehydrogenase [82]. The increased number of GH43 and auxiliary functions indicate the ability of endophytes to degrade lignocellulose. The much greater number of CAZymes in the genomes of endophytes showed that endophytic fungi use different carbohydrate uptake systems than pathogenic fungi to adapt to symbiotic environmental conditions.

In recent years, SSPs have been extensively studied as effectors that are involved in host-pathogen interactions [83]. These SSPs are found in most fungal species regardless of their nutritional lifestyles. Interestingly, the number of SSPs of endophytic fungi is much higher than that of pathogens, indicating that SSPs might be related to the colonization of endophytic fungi in plants and the promotion of nutritional dominance, which is consistent with previous studies [55,84,85].

The number of identified secondary metabolite gene clusters in the genomes of AS31, AS33, and AS42 was substantially high, suggesting a large and yet-to-be-identified metabolic potential. These compounds may have functions in changing plant behavior [86]. For example, the three strains have more indole clusters than pathogens. Given the importance of indole metabolism in the synthesis of auxin (indole-3-acetic acid), the results showed the important roles of indole metabolism in endophyte-plant interactions. In addition, the three endophytes all possess gene clusters involved in the synthesis of asperthecin. The ascosporous pigment of *A. nidulans* is reportedly formed by asperthecin, and the asperthecin biosynthetic gene cluster consists of three genes, aptA, aptB, and aptC, in which the deletion of aptA (encoding polyketide synthase) or aptB (encoding thioesterase) produces small, malformed transparent ascospores, whereas the deletion of aptC (encoding a monooxygenase) produces morphologically normal but purple ascospores [87]. These results suggested that the asperthecin biosynthetic gene clusters of the three endophytes are important to maintaining normal spore morphology and successfully colonizing rice roots.

## 5. Conclusions

In this study, endophytes belonging to *Aspergillus* that could significantly promote the growth of rice were isolated. The beneficial interactions between the endophytes and host plants were explored by genomic sequencing and comparative genome analysis. Our results showed that the genomes of the endophytes had more CAZymes, SSPs, and indole clusters than those of the pathogens, which could be involved in endophytism. Specifically, CAZymes and SSPs could help to adapt to symbiotic environmental conditions by using different carbon sources and addressing plant defense responses, while indole and asperthecin biosynthetic genes are functional in maintaining colonization and promoting plant growth. Additionally, the endophytes induced the expression of Pi transport genes in rice and improved Pi absorption and transport in rice. These results help us to understand the endophyte-plant interactions and growth-promoting mechanism of endophytes, paving the way for the practical application of endophytic fungi in agriculture.

## Figures and Tables

**Table 1:** Genome features of strains AS31, AS33, and AS42.

	AS31	AS33	AS42
Accession number	JAIOTV000000000	JAIOTX000000000	JAIOTW000000000
Number of scaffolds	538	812	836
Genome size (Mb)	36.8	34.8	35.3
N50 length (bp)	246,959	284,941	76,567
GC content (%)	49.94	49.57	50.47
N rate (%)	0	0	0.0001
Gene number	12,933	12,364	13,211
Gene average length (bp)	1474	1514	1453
Gene length/Genome (%)	53.85	47.48	54.87
Repeat (%)	2.28	1.62	6.43
NR	12,759	12,685	12,949
GO	4093	4117	4128
eggNOG	7732	7864	7852
KEGG	4644	4684	4612
Swiss	7667	7829	7745

## Data Availability

The data presented in this study are openly available in NCBI, BioProject number [PRJNA757213]. Publicly available datasets were analyzed in this study. This data can be found here: [*A. sydowii* (PRJNA721994); *A. versicolor* (PRJNA721993); *A. awamori* (PRJDB4986); *A. nidulans* (PRJNA13961); *A. ochraceoroseus* (PRJNA275128); *A. tubingensis* (PRJNA645154); *A. puulaauensis* (PRJNA728012)].

## Supplementary Materials

The following supporting information can be downloaded at: https://www.mdpi.com/article/10.3390/jof8070690/s1, Figure S1: The classifications of genes of AS31, AS33, and AS42 based on KEGG categories. A, Metabolism; B, Genetic information processing; C, Environmental information processing; D, Cellular processes; E, Organismal systems; Figure S2: The top 20 KEGG pathway enriched in genomes of AS31, AS33, and AS42; Figure S3: GO function classification of genes in the genomes of AS31, AS33, and AS42; Figure S4: Venn diagrams showing the annotated genes in the genomes of AS31, AS33, and AS42 based on databases including NR, Swissport, COG, GO and KEGG; Table S1: Sequences of the primers used in qPCR; Table S2: The expression of Pi transport-related genes. Table S3: Statistics of genome assemblies of selected Aspergillus strains used in this study; Table S4: Numbers of secondary metabolism gene clusters in genomes of AS31, AS33, and AS42; Table S5: Secondary metabolite gene clusters of AS31; Table S6: Secondary metabolite gene clusters of AS33; Table S7: Secondary metabolite gene clusters of AS42.
