# Supplementary material for: Comparative Genomics of Three Aspergillus Strains Reveals Insights into Endophytic Lifestyle and Endophyte-Induced Plant Growth Promotion
Source: J Fungi (Basel). 2022 Jun 29;8(7):690. doi: 10.3390/jof8070690 (PMC9323082; doi:10.3390/jof8070690)
Supplement: Supplementary file 1 [file jof-08-00690-s001.zip › jof-1760361-supplementary.pdf]

Supplementary Figures

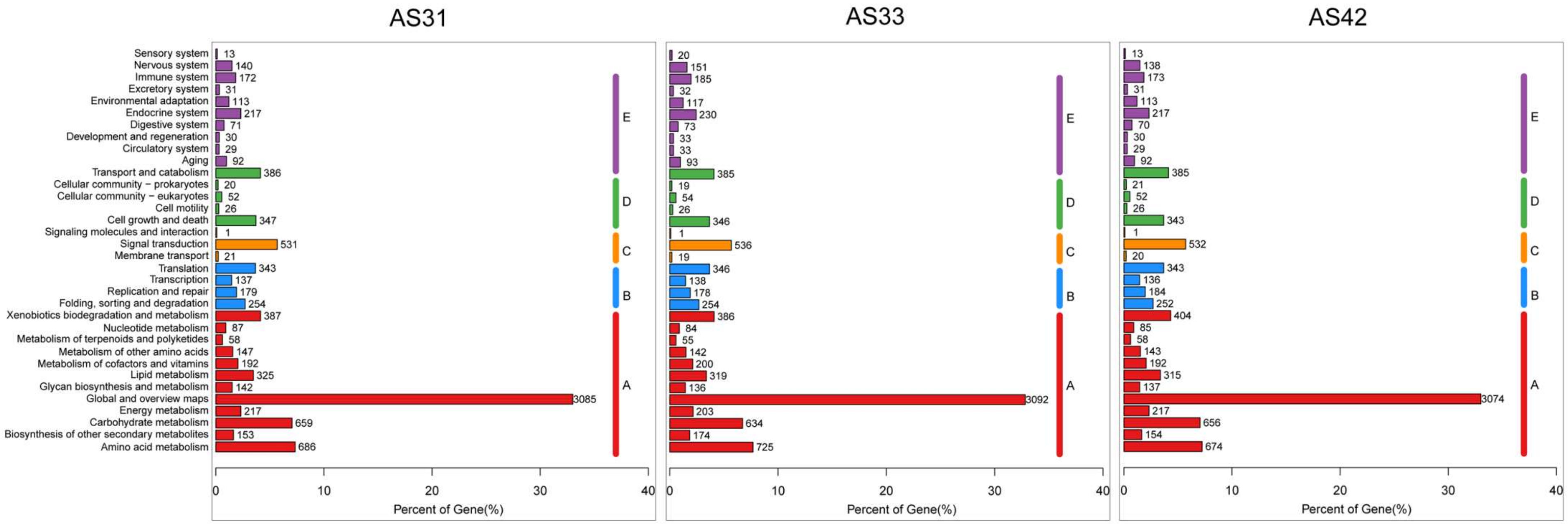

Figure S1. The classifications of genes of AS31, AS33, and AS42 based on KEGG categories. A, Metabolism; B, Genetic information processing; C, Environmental information processing; D, Cellular processes; E, Organismal systems.

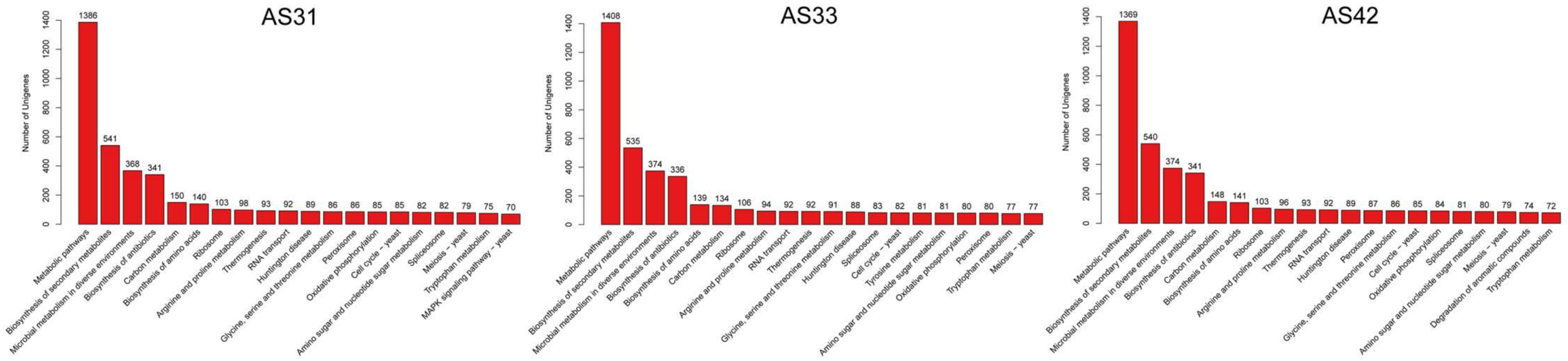

Figure S2. The top 20 KEGG pathways enriched in genomes of AS31, AS33, and AS42.

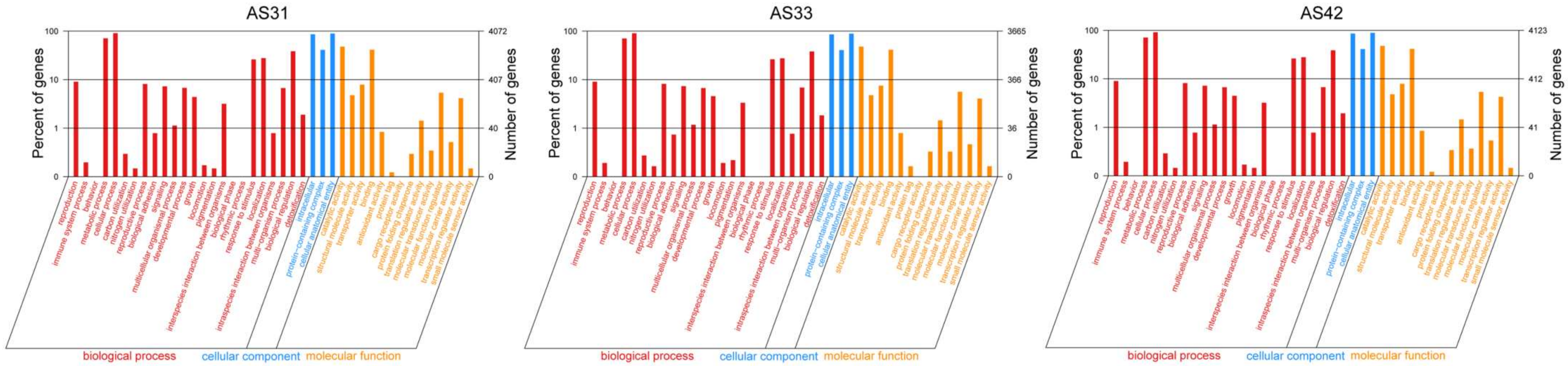

Figure S3. GO function classification of genes in the genomes of AS31, AS33, and AS42.

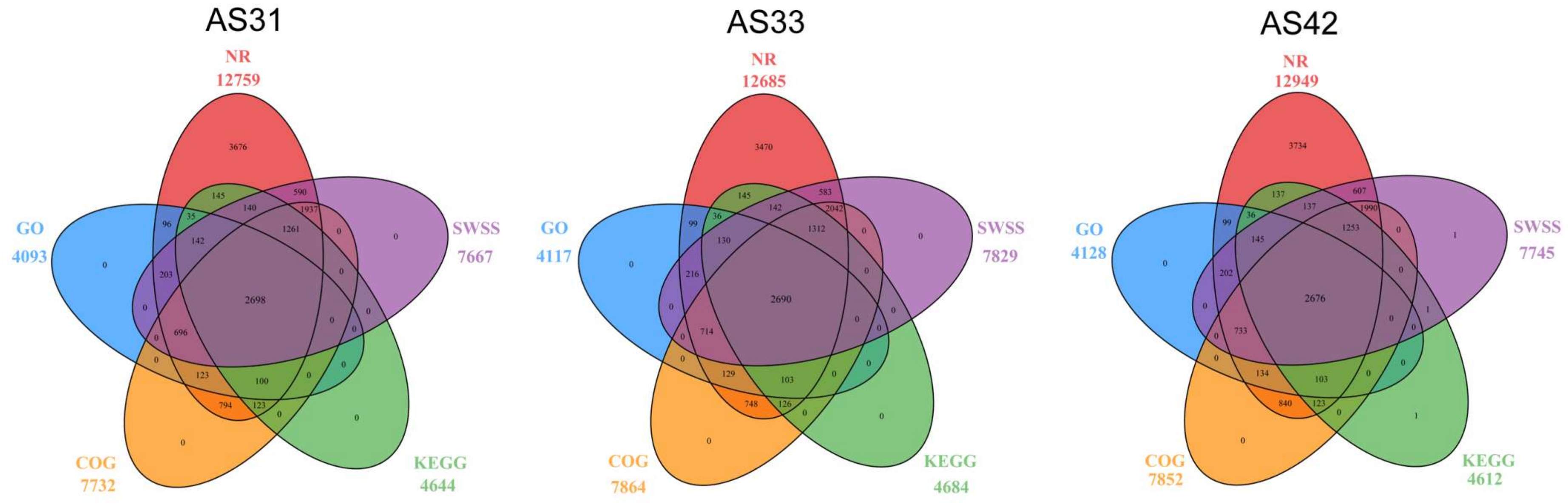

Figure S4. Venn diagrams showing the annotated genes in the genomes of AS31, AS33, and AS42 based on databases including NR, Swissport, COG, GO and KEGG.

Supplementary Tables

Table S1. Sequences of the primers used in qPCR.

Primers	Sequence (5'-3')
actin-F	TTATGGTTGGGATGGGACA
actin-R	AGCACGGCTTGAATAGCG
OsPT1-F	CGCTTCCGTACGAGTGGTAGT
OsPT1-R	GGTTCTTTCAAATCCAGGGAAA
OsPT2-F	GACGAGACCGCCCAAGAAG
OsPT2-R	TTTTCAGTCACTCACGTCGAGAC
OsPT4-F	TATTGCGGCTTAGATTGCATTAG
OsPT4-R	TCCAAATCAAATGGGCACTAAG
OsPT5-F	TGCTACTGCCCATGACTAGGATT
OsPT5-R	CCATAGAAGAGATCCAGAGAAGCTGTA
OsPT6-F	TATAACTGATCGATCGAGACCAGAG
OsPT6-R	TGGATAGCCAGGCCAGTTATATATC
OsPT7-F	GCAAGTCGCTCGAGGAGATG
OsPT7-R	TGGAATTAACGGGTGGATCAC
OsPT8-F	AGAAGGCAAAAGAAATGTGTGTTAAAT
OsPT8-R	AAAATGTATTCGTGCCAAATTGCT
OsPT9-F	CATAGGCTTGTCATCCTTTGG
OsPT9-R	CACTGTAAATAAATCCGCGTTTC
OsPT10-F	GAGCTCGCACCTCAGCAT
OsPT10-R	GAGTTCACTCACACGGAGACC
OsIPS1-F	AAGGGCAGGGCACACTCCACATTA
OsIPS1-R	ATTAGAGCAAGGACCGAAACACA
OsSPX1-F	CTGAAAACGGTAATGGATAGG
OsSPX1-R	AACAACAACAGCACGAGC
OsPAP9b-F	ACCTACGATAACAGCAACTACGC
OsPAP9b-R	CATATCAGTTTTCGTCGCATGTA
OsPT11-F	CATATCCCAGATGAGCGTATCATG
OsPT11-R	GAGAAGTTCCCTGCTTCAAGCA
OsPT13-F	CTTCTCCATCTCCCTTGTCG
OsPT13-R	TTTTGTCGCCTAGCCAGCC

Table S2. The expression of Pi transport-related genes in roots and shoots of rice at 7 and 14 days after inoculation with strains AS31, AS33, or AS42. The gene expression (Ct value) was obtained by qRT-PCR.

	β-actin	PT1	PT2	PT4	PT5	PT6	PT7	PT8	PT9	PT10	IPS1	SPX1	PAP9b	PT11	PT13
Root-CK-7days	33.10±0.045	31.49±0.39	33.54±0.094	30.89±0.68	33.93±0.61	37.02±0.11	36.89±0.42	27.71±0.41	33.87±0.069	36.84±0.55	33.30±0.26	32.18±0.43	32.64±0.46	35.74±0.012	35.83±0.70
Shoot-CK-7days	26.74±0.34	22.68±0.28	29.39±0.83	33.08±0.65	28.78±0.60	35.41±0.86	35.12±0.36	23.18±0.38	35.71±0.45	33.22±0.46	25.63±0.69	23.12±0.18	25.81±0.53	32.72±0.67	34.70±0.16
Root-AS31-7days	33.22±0.54	29.02±0.060	29.81±0.41	29.08±0.59	35.44±0.25	35.53±0.31	38.574±0.42	29.21±0.38	36.45±0.16	34.61±0.14	36.19±0.27	34.73±0.28	35.20±0.047	37.09±0.033	35.45±0.23
Shoot-AS31-7days	26.46±0.25	21.43±0.19	28.18±0.42	33.90±0.45	28.11±0.11	37.13±0.057	36.49±0.074	25.03±0.090	35.24±0.17	34.71±0.49	29.27±0.002	25.00±0.83	29.54±0.29	35.64±0.046	37.08±0.043
Root-AS33-7days	33.86±0.36	29.41±0.34	34.00±0.35	33.18±0.41	35.04±0.062	34.67±0.56	34.51±0.54	27.81±0.29	36.11±0.076	30.21±0.19	31.06±0.35	29.28±0.13	35.83±0.20	27.68±0.036	34.72±0.19
Shoot-AS33-7days	26.28±0.50	20.98±0.14	24.09±0.22	33.91±0.005	27.78±0.27	37.07±0.068	35.84±0.63	24.50±0.74	33.15±0.59	34.23±0.25	25.23±0.058	26.09±0.016	28.14±0.48	34.91±0.41	35.54±0.026
Root-AS42-7days	34.55±0.52	31.29±0.14	33.96±0.39	34.06±0.16	36.99±0.003	36.93±0.21	35.96±0.15	31.25±0.20	36.88±0.25	35.38±0.29	33.51±0.66	32.37±0.25	35.52±0.65	27.99±0.45	35.08±0.26
Shoot-AS42-7days	27.96±0.57	25.02±0.021	30.30±0.087	34.50±0.13	30.63±0.37	36.86±0.42	36.52±0.20	27.73±0.72	36.10±0.077	35.38±0.20	27.79±0.56	27.69±0.28	29.45±0.097	36.20±0.18	36.66±0.10
Root-CK-14days	15.64±0.27	11.71±0.30	17.75±0.035	15.76±0.20	15.47±0.52	21.44±0.28	18.14±0.36	11.96±0.47	19.63±0.38	18.93±0.069	18.10±0.07	16.89±0.20	16.06±0.042	19.57±0.34	19.80±0.13
Shoot-CK-14days	18.87±0.016	16.20±0.34	19.92±0.016	20.10±0.38	20.96±0.47	21.44±0.075	20.25±0.47	17.18±0.46	23.36±0.43	21.41±0.38	22.59±0.012	21.61±0.61	20.02±0.37	24.93±0.007	21.93±0.36
Root-AS31-14days	28.64±0.20	24.15±0.25	31.21±0.008	24.43±0.071	28.43±0.13	34.92±0.47	33.31±0.35	23.99±0.23	31.40±0.45	32.52±0.14	29.52±0.31	30.53±0.27	28.63±0.44	33.49±0.42	32.70±0.26
Shoot-AS31-14days	30.86±0.57	26.18±0.18	34.70±0.031	31.68±0.086	32.13±0.14	35.01±0.37	33.89±0.039	26.64±0.24	34.87±0.37	33.77±0.24	33.42±0.19	32.12±0.043	30.30±0.097	35.14±0.41	34.46±0.016
Root-AS33-14days	27.25±0.16	23.18±0.17	28.59±0.39	28.27±0.25	28.35±0.37	31.82±0.40	33.02±0.31	23.48±0.40	28.73±0.06	32.47±0.62	28.86±0.081	29.15±0.022	28.03±0.13	34.67±0.28	32.37±0.42
Shoot-AS33-14days	29.23±0.043	23.63±0.11	31.23±0.46	31.08±0.17	31.29±0.59	35.79±0.37	33.80±0.062	27.16±0.11	37.10±0.49	33.73±0.25	31.26±0.053	28.78±0.48	28.22±0.47	36.38±0.071	34.12±0.46
Root-AS42-14days	30.98±0.37	25.12±0.22	31.65±0.35	31.45±0.13	33.69±0.54	32.88±0.001	32.38±0.25	27.38±0.14	32.81±0.47	31.15±0.14	32.30±0.064	30.50±0.033	31.75±0.45	36.71±0.079	33.56±0.16
Shoot-AS42-14days	34.66±0.48	25.23±0.041	35.39±0.46	32.98±0.32	35.04±0.26	35.61±0.053	35.02±0.50	27.81±0.13	38.65±0.52	35.47±0.044	33.26±0.044	33.55±0.22	32.46±0.093	38.79±0.41	33.88±0.49

Table S3. Statistics of genome assemblies of selected *Aspergillus* strains used in this study.

Strains	BioProjects	Number of Scaffolds	N50 (bp)	Total length (Mb)	Protein counts	GC (%)
Aspergillus sydowii CBS 593.65	PRJNA721994	97	123783	34.4	13579	50.0
Aspergillus versicolor CBS 583.65	PRJNA721993	51	695,565	33.1	13222	50.1
Aspergillus awamori	PRJDB4986	33	4298649	38.6	11224	49.3
AS31	PRJNA 757213	538	246959	36.8	12,933	49.9
AS33	PRJNA 757213	812	284941	34.8	12,898	49.6
AS42	PRJNA 757213	836	76567	35.3	13,211	50.6
Aspergillus nidulans FGSC A4	PRJNA13961	91	282268	30.3	9556	50.3
Aspergillus ochraceoroseus SRRC1432	PRJNA275128	3590	21535	24.3	7829	48.2
Aspergillus tubingensis WU-2223L	PRJNA645154	15	3153774	35.0	11477	49.3
Aspergillus puulaauensis MK2	PRJNA728012	8	4388637	34.3	13620	49.754

Table S4. Numbers of secondary metabolism gene clusters in genomes of AS31, AS33, and AS42.

Strains	NRPS	T1PKS	T3PKS	terpene	fungal-RiPP	betalactone	indole	siderophore	NAPAA	Total
Aspergillus sydowii CBS 593.65	23	20	0	9	1	3	6	0	0	56
AS31	28	21	0	10	1	3	7	0	0	60
AS33	34	23	0	11	1	3	8	0	0	68
AS42	27	18	0	10	1	3	7	0	0	58
Aspergillus versicolor CBS 583.65	23	15	0	6	0	3	4	0	0	43
Aspergillus awamori	39	30	1	7	0	1	2	0	0	70
Aspergillus nidulans FGSC A4	24	22	0	9	0	3	4	0	0	55
Aspergillus ochraceoroseus SRRC1432	13	13	0	6	0	1	4	0	0	34
Aspergillus tubingensis WU-2223L	39	38	1	21	0	1	2	2	0	83
Aspergillus puulaauensis MK2	33	25	0	11	0	2	8	0	1	67

Table S5. Secondary metabolite gene clusters of AS31.

Cluster	Region	Type	Location	Most similar known cluster	Similarity
cluster1	Region 1.1	fungal-RiPP	10,736-45,020	unknown	
	Region 1.2	indole	106,415-127,722	unknown	
	Region 1.3	T1PKS	538,544-585,245	naphthopyrone	100%
	Region 1.4	terpene	706,380-728,735	clavaric acid	100%
cluster4	Region 4.1	NRPS-like	161,194-204,016	unknown	
cluster6	Region 6.1	T1PKS	37,472-83,035	neosartorin	36%
cluster7	Region 7.1	NRPS-like	171,628-207,102	unknown	
	Region 7.2	NRPS	374,347-428,667	unknown	
cluster8	Region 8.1	NRPS-like	81,035-125,551	unknown	
cluster9	Region 9.1	T1PKS	25,621-72,939	4-epi-15-epi-brefeldin A	20%
cluster10	Region 10.1	terpene	416,122-437,706	squalestatin S1	80%
	Region 10.2	T1PKS	440,145-470,244	unknown	
cluster11	Region 11.1	T1PKS	154,467-202,345	fusaridione A	12%
cluster13	Region 13.1	terpene	119,573-140,630	unknown	
cluster15	Region 15.1	NRPS	217,956-266,666	penicillin	18%
	Region 15.2	NRPS	285,972-330,066	unknown	
cluster16	Region 16.1	terpene	30,219-51,181	unknown	
	Region 16.2	NRPS-like,T1PKS,NRPS	146,463-238,685	leucinostatin A / leucinostatin B	10%
cluster18	Region 18.1	NRPS-like	170,998-214,287	burnettramic acid A	22%
cluster22	Region 22.1	NRPS-like	316,175-354,441	unknown	
cluster23	Region 23.1	terpene	80,087-102,135	unknown	
cluster25	Region 25.1	T1PKS,terpene	291,960-329,950	shearinine D	9%
cluster30	Region 30.1	T1PKS	193,949-239,515	asperthecin	100%
cluster33	Region 33.1	indole	150,050-171,428	unknown	
cluster34	Region 34.1	NRPS	33,753-86,067	fellutamide B	100%
cluster38	Region 38.1	T1PKS	107,129-154,242	unknown	
	Region 38.2	T1PKS	234,143-267,505	F9775A / F9775B / orsellinic acid	60%
cluster41	Region 41.1	T1PKS,NRPS-like	51,960-106,698	yanuthone D	20%
cluster45	Region 45.1	NRPS	52,849-107,051	unknown	
	Region 45.2	indole	212,733-234,099	unknown	
cluster47	Region 47.1	T1PKS	110,103-157,857	unknown	
cluster48	Region 48.1	NRPS,betalactone	151,907-197,043	unknown	
cluster49	Region 49.1	NRPS-like,indole	201,771-228,220	ochrindole A	29%
cluster52	Region 52.1	NRPS-like	163,731-207,000	unknown	
cluster54	Region 54.1	NRPS-like	82,641-125,921	unknown	
cluster57	Region 57.1	NRPS	65,420-124,473	nidulanin A	75%
cluster61	Region 61.1	T1PKS,NRPS	69,384-121,526	pyranonigrin E	100%

cluster62	Region 62.1	terpene	90,331-112,3377	unknown	
	Region 62.2	T1PKS,indole	159,109-196,515	neosartoricin B	60%
cluster65	Region 65.1	NRPS-like	139,759-184,646	unknown	
cluster74	Region 74.1	terpene	1-14,271	unknown	
cluster79	Region 79.1	terpene	44,807-65,919	unknown	
cluster81	Region 81.1	NRPS-like	114,978-156,127	unknown	
cluster84	Region 84.1	NRPS	26,003-73,316	unknown	
cluster89	Region 89.1	NRPS,T1PKS	64,819-135.028	unknown	
cluster90	Region 90.1	NRPS	28,910-76,575	unknown	
cluster91	Region 91.1	T1PKS	95,328-133,961	neosartorin	31%
cluster93	Region 93.1	NRPS,T1PKS	1-41,591	equisetin	54%
cluster96	Region 96.1	T1PKS	1-37,693	asperfuranone	18%
cluster97	Region 97.1	indole	82,454-103,656	unknown	
cluster98	Region 98.1	terpene,NRPS-like	67,218-118,691	clavaric acid	100%
cluster101	Region 101.1	indole	23,998-45,431	unknown	
cluster113	Region 113.1	betalactone	36,569-67,108	unknown	
cluster126	Region 126.1	betalactone	16,258-40,236	unknown	
cluster130	Region 130.1	NRPS	1-23,896	unknown	
cluster154	Region 154.1	T1PKS	27,679-61,601	unknown	
cluster157	Region 157.1	NRPS	1-50,534	nidulanin A	50%
cluster158	Region 158.1	T1PKS	1-39,626	unknown	
cluster161	Region 161.1	NRPS-like	28,617-55,428	unknown	
cluster181	Region 181.1	T1PKS	1-37,835	unknown	

Table S6. Secondary metabolite gene clusters of AS33.

Cluster	Region	Type	Location	Most similar known cluster	Similarity
cluster1	Region 1.1	T1PKS	136,958-180,054	unknown	
	Region 1.2	terpene	344,848-365,958	unknown	
	Region 1.3	NRPS,indole	470,547-517,263	notoamide A	66%
	Region 1.4	T1PKS	678,772-727,593	unknown	
cluster2	Region 2.1	terpene,NRPS-like	561,618-613,189	clavaric acid	100%
cluster3	Region 3.1	T1PKS	70,151-115,767	neosartorin	36%
	Region 3.2	terpene	418,308-439,499	unknown	
cluster4	Region 4.1	NRPS	260,937-313,249	fellutamide B	100%
cluster5	Region 5.1	betalactone	64,969-94839	unknown	
cluster6	Region 6.1	NRPS-like	203,183-247,674	unknown	
cluster7	Region 7.1	NRPS,T1PKS	43,955-134,293	sterigmatocystin	65%
	Region 7.2	indole	254,454-275,793	unknown	
cluster9	Region 9.1	NRPS	153,720-203,535	unknown	
cluster10	Region 10.1	T1PKS,NRPS	368,831-413,643	pyranonigrin E	100%
cluster11	Region 11.1	NRPS-like,T1PKS	1-55,256	unknown	
	Region 11.2	T1PKS	303,715-351,802	unknown	
cluster13	Region 13.1	terpene	197,585-219,171	squalestatin S1	80%
	Region 13.2	T1PKS	22,1647-269,298	unknown	
cluster14	Region 14.1	NRPS	53,856-109,944	unknown	
cluster15	Region 15.1	T1PKS	33,227-90,743	chaetoviridin E / 11-epichaetomugilin A	11%
cluster18	Region 18.1	NRPS	92,119-140,659	penigequinolone A	21%
cluster21	Region 21.1	T1PKS	127,614-173,181	asperthecin	100%
cluster22	Region 22.1	NRPS	253,925-312,973	nidulanin A	75%
cluster23	Region 23.1	NRPS-like,indole	78,217-121,108	ochrindole A	29%
cluster24	Region 24.1	NRPS	229,404-28,679	cyclo-(D-Phe-L-Phe-D-Val-L-Val)	100%
cluster25	Region 25.1	T1PKS	108,305-155,812	neurosporin A	26%
cluster28	Region 28.1	T1PKS	64,443-111,370	naphthopyrone	100%
cluster30	Region 30.1	NRPS-like	1-30,186	unknown	
cluster31	Region 31.1	NRPS-like	45,718-90,602	unknown	
cluster34	Region 34.1	betalactone	72,138-96,121	unknown	
	Region 34.2	NRPS,NRPS-like	100,504-183,552	unknown	
cluster37	Region 37.1	T1PKS,terpene	1-38,199	shearinine D	9%
cluster41	Region 41.1	indole	151,788-173,182	unknown	
cluster42	Region 42.1	NRPS,betalactone	116,158-161,288	unknown	
cluster43	Region 43.1	indole	168,615-189,989	unknown	
	Region 43.2	NRPS-like	232,938-266,294	unknown	
cluster45	Region 45.1	terpene	128,622-149,584	unknown	
cluster46	Region 46.1	T1PKS	101,804-149,818	unknown	
	Region 46.2	terpene	236,993-254,079	unknown	

cluster47	Region 47.1	NRPS-like	189,258-232,565	unknown	
cluster48	Region 48.1	NRPS,terpene	89,959-133,635	unknown	
	Region 48.2	NRPS	193,476-246,043	unknown	
cluster49	Region 49.1	NRPS-like	6,305-49,346	unknown	
cluster51	Region 51.1	T1PKS	172,096-220,189	ankaflavin / monascin / rubropunctatine / monascorubrin	20%
cluster56	Region 56.1	NRPS	67,739-122,054	unknown	
cluster60	Region 60.1	NRPS,NRPS-like	1-72,089	unknown	
cluster63	Region 63.1	terpene	163,604-184,697	unknown	
cluster68	Region 68.1	terpene	138,943-160,077	shearinine D	13%
cluster73	Region 73.1	NRPS-like	41,739-85,070	unknown	
cluster76	Region 76.1	T1PKS	15,170-64,943	unknown	
cluster79	Region 79.1	NRPS-like	76,600-120,066	unknown	
cluster86	Region 86.1	NRPS,T1PKS	22,307-73,332	asperfuranone	18%
cluster93	Region 93.1	indole	119,160-140,600	unknown	
cluster96	Region 96.1	terpene	57,677-80,032	unknown	
cluster101	Region 101.1	fungal-RiPP	1-27,095	unknown	
cluster103	Region 103.1	NRPS-like	1-25,046	unknown	
cluster104	Region 104.1	NRPS-like	1-24,584	unknown	
cluster105	Region 105.1	T1PKS,NRPS-like	20,621-75,269	yanuthone D	20%
cluster109	Region 109.1	indole	28,976-50,423	unknown	
cluster125	Region 125.1	NRPS	45,844-94,585	unknown	
cluster144	Region 144.1	NRPS	34,163-74,230	unknown	
cluster156	Region 156.1	T1PKS	1-33,857	unknown	
cluster164	Region 164.1	NRPS	1,282-52,405	aspercryptins	40%
cluster165	Region 165.1	T1PKS	1-32,651	F9775A / F9775B / orsellinic acid	60%
cluster175	Region 175.1	indole	12,436-33,714	unknown	
cluster180	Region 180.1	T1PKS	1-37,437	unknown	
cluster201	Region 201.1	NRPS,T1PKS	1-29,623	burnettramic acid A	88%
cluster208	Region 208.1	T1PKS	1-27,318	pyripyropene A	33%

Table S7. Secondary metabolite gene clusters of AS42.

cluster	Region	Type	location	Most similar known cluster	Similarity
cluster1	Region 1.1	fungal-RiPP	59,154-93,438	unknown	
	Region 1.2	indole	154,944-176,251	unknown	
cluster2	Region 2.1	NRPS	1-33,436	unknown	
cluster3	Region 3.1	NRPS	174,627-221,940	unknown	
cluster5	Region 5.1	T1PKS	12,036-55,562	unknown	
	Region 5.2	NRPS,T1PKS	103,759-192,476	unknown	
cluster6	Region 6.1	NRPS,T1PKS	1-41,592	equisetin	54%
cluster8	Region 8.1	NRPS,betalactone	39,486-84,622	unknown	
cluster9	Region 9.1	T1PKS,NRPS-like	127,241-181,979	yanuthone D	20%
cluster11	Region 11.1	NRPS	1-39,028	unknown	
	Region 11.2	indole	144,710-166,076	unknown	
cluster16	Region 16.1	indole	151,868-173,247	unknown	
cluster24	Region 24.1	terpene	105,705-126,817	unknown	
cluster25	Region 25.1	NRPS-like	5,378-48,290	unknown	
cluster26	Region 26.1	NRPS-like	46,238-90,754	unknown	
cluster29	Region 29.1	NRPS-like	123,451-161,630	unknown	
cluster38	Region 38.1	T1PKS	43,150-88,713	neosartorin	36%
cluster41	Region 41.1	NRPS-like	48,582-93469	unknown	
cluster42	Region 42.1	terpene,T1PKS	70,675-116,504	shearinine D	9%
cluster48	Region 48.1	T1PKS	48,151-96,029	fusaridione A	12%
cluster49	Region 49.1	T1PKS	74,253-119,757	neosartorin	42%
cluster50	Region 50.1	T1PKS	1-46,098	naphthopyrone	100%
cluster54	Region 54.1	T1PKS	1-29,498	unknown	
cluster58	Region 58.1	indole	12,710-34143	unknown	
cluster69	Region 69.1	NRPS	28,471-79,753	penicillin	18%
cluster81	Region 81.1	NRPS	60,816-103,996	fellutamide B	100%
cluster90	Region 90.1	NRPS,T1PKS,NRPS-like	17,047-96,458	leucinostatin A / leucinostatin B	10%
cluster96	Region 96.1	T1PKS	21,875-67,441	asperthecin	100%
cluster98	Region 98.1	terpene	69,645-91,682	unknown	
cluster100	Region 100.1	indole	1-14,567	unknown	
	Region 100.2	NRPS-like	56,548-90,019	unknown	
cluster106	Region 106.1	NRPS	45,099-89,098	unknown	
cluster107	Region 107.1	terpene	17,628-39,634	unknown	
cluster117	Region 117.1	T1PKS	49,327-82,973	F9775A / F9775B / orsellinic acid	60%
cluster120	Region 120.1	NRPS-like	1-41,941	unknown	
cluster123	Region 123.1	T1PKS,indole	7,612-53,161	neosartoricin B	60%
cluster126	Region 126.1	NRPS	43,210-78,659	unknown	
cluster142	Region 142.1	betalactone	31,751-62,290	unknown	
cluster163	Region 163.1	NRPS-like	1-38,268	unknown	
cluster178	Region 178.1	betalactone	43,576-66,679	unknown	

cluster180	Region 180.1	NRPS-like	28,232-66,206	unknown	
cluster186	Region 186.1	NRPS-like	24,053-64,395	unknown	
cluster217	Region 217.1	terpene	8,068-30,423	clavaric acid	100%
cluster226	Region 226.1	T1PKS	28,459-56,069	unknown	
cluster248	Region 248.1	NRPS-like	27,681-49,936	unknown	
cluster305	Region 305.1	T1PKS	1-39,998	unknown	
cluster311	Region 311.1	NRPS-like,terpene	1-39,251	clavaric acid	100%
cluster317	Region 317.1	terpene	1-12,755	unknown	
cluster345	Region 345.1	indole	22,767-34,159	terrequinone A	80%
cluster347	Region 347.1	T1PKS	1-26,867	pyranonigrin E	100%
cluster368	Region 368.1	NRPS-like	7,324-31,106	unknown	
cluster428	Region 428.1	NRPS	1-23,715	nidulanin A	50%
cluster440	Region 440.1	NRPS	1-22,472	cyclo-(D-Phe-L-Phe-D-Val-L-Val)	100%
cluster488	Region 488.1	terpene	1-16,530	unknown	
cluster504	Region 504.1	terpene	694-15,942	squalestatin S1	80%
cluster522	Region 522.1	NRPS	1-14,562	nidulanin A	50%
cluster569	Region 569.1	T1PKS	1-11,499	unknown	
cluster672	Region 672.1	terpene	1-6,210	unknown	
